# How Much Energy Do E’Athletes Use during Gameplay? Quantifying Energy Expenditure and Heart Rate Variability Within E’Athletes

**DOI:** 10.1186/s40798-024-00708-6

**Published:** 2024-04-17

**Authors:** Mitchell Nicholson, Dylan Poulus, Rob Robergs, Vincent Kelly, Craig McNulty

**Affiliations:** 1 School of Exercise and Nutrition Sciences, Faculty of Health, Queensland University of Technology (QUT), Victoria Park Road, Kelvin Grove, Brisbane, QLD 4059 Australia; 2 Physical Activity, Sport and Exercise Research Theme, Faculty of Health, Southern Cross University, Gold Coast, QLD Australia; 3 Manna Institute, Southern Cross University, Gold Coast, Australia

**Keywords:** Esports, Metabolism, Physiology, Heart rate variability, Indirect calorimetry, Gas analysis

## Abstract

**Background:**

Research into esports suggests that e’athletes experience physiological stressors and demands during competition and training. The physiological demands of esports are poorly understood and need to be investigated further to inform future training guidelines, optimise performance outcomes, and manage e’athlete wellbeing. This research aimed to quantify the metabolic rate of esports gameplay and compare this outcome with heart rate variability within expert e’athletes.

**Results:**

Thirteen healthy male participants ranked within the top 10% of their respective esports title participated in the study (age = 20.7 ± 2.69 years; BMI = 24.6 ± 5.89 kg·m^− 2^). Expired gas analysis indirect calorimetry measured gas exchange during rest and gaming. Compared to resting conditions, competitive esports gameplay significantly increased median energy expenditure (1.28 (IQR 1.16–1.49) kcal·min^− 1^ vs. 1.45 (IQR 1.20–1.77) kcal·min^− 1^, *p* = .02), oxygen consumption (0.27 (IQR 0.24–0.30) L·min^− 1^ vs. 0.29 (IQR 0.24–0.35) L·min^− 1^, *p* = .02) and carbon dioxide production (0.20 (IQR 0.19–0.27) L·min^− 1^vs. 0.27 (IQR 0.24–0.33) L·min^− 1^, *p* = .01). Competitive gameplay also resulted in a significant increase in heart rate (84.5 (IQR 74.1–96.1) bpm vs. 87.1 (IQR 80.3–104) bpm, *p* = .01) and decrease in R-R interval’s (710 (IQR 624–810) ms vs. 689 (IQR 579–747) ms, *p* = .02) when compared to rest. However, there were no significant differences in time or frequency measures of heart rate variability.

**Conclusions:**

The data reveal increased physiological responses to metabolic rate, energy expenditure and cardiovascular function to esports game play within expert e’athletes. Further physiological research into the physical demands on e’athletes, the influence of different training programs to esport performance, and the added multivariate determinants to elite level esport performance are warranted.

**Supplementary Information:**

The online version contains supplementary material available at 10.1186/s40798-024-00708-6.

## Background

Competitive esports athletes (e’athletes; [1]) face stressors and demands within training and competition, both psychologically and physiologically [2–4]. Due to the highly competitive, and sometimes lucrative, nature of esports, various psychophysiological stress responses under competitive conditions may be experienced [5]. Esport performance does not involve large bodily movements; rather, e’athletes require fine-motor coordination and perceptual-cognitive abilities to perform at a high level [5]. Preliminary research into the structure of training within esports has identified no specific training guidelines or adoption of player monitoring [6]. Comparatively, traditional sport settings quantify various internal (e.g., heart rate (HR), oxygen uptake (V̇O_2_), blood lactate, perceived exertion, etc.) and external (e.g., running distances, power output, speed, repetitions, etc.) stressors or loads within athletes to monitor how individuals are responding to the demands of training or competition to optimise health and performance [7]. Despite the rapid increase in popularity of esports, the physiological demands and stressors of esport are poorly understood [3, 8]. By investigating the physiological demands of esports, insights could be identified that may inform training guidelines to optimise performance and the health and wellbeing of e’athletes.

Expired gas analysis indirect calorimetry (EGAIC) is considered the gold-standard in measuring human energy expenditure through pulmonary gas exchange [9, 10]. The application of calorimetry in sport and medical physiology often utilises EGAIC to determine individual energy expenditure by measuring the rate of oxygen consumption (V̇O_2_) and carbon dioxide production (V̇CO_2_) within various settings and activities. Dividing V̇CO_2_ with V̇O_2_ yields the respiratory exchange ratio (RER), which for conditions of steady state metabolism and normal ventilation enables the calculation of energy expenditure and each of carbohydrate and fat oxidation [11, 12] based on the higher CO2 production for aerobic (mitochondrial respiration) carbohydrate oxidation. For example, at rest or steady state exercise the body will metabolise predominantly fat through cellular respiration (RER for pure fat metabolism = 0.7 and 4.73 kcals·L^− 1^ V̇O_2_), although as exercise or activity intensity increases, the body will shift to metabolising carbohydrates (RER for pure carbohydrate oxidation = 1.0 and 5.05 kcals·L^− 1^ V̇O_2_)[11, 12].

Previous research has shown that the energy expenditure of sedentary activities (e.g., reading, typing, watching TV, etc.) is ≤ 1.5 metabolic equivalents (METs), where one MET is equivalent to an average resting V̇O_2_ of 3.5 ml·kg^−1^·min^−1^ within healthy individuals [13, 14]. While esports involves sitting, the increased movement and cognitive demand distinguish them from traditional sedentary activities. Most research has focused on comparing active video gaming energy expenditure research (e.g., Wii Sports, XBOX Kinect, or Virtual Reality, that is played standing) and casual video gaming (e.g., controller or keyboard and mouse-based that is sedentary), with conflicting findings across settings [15]. Two studies have shown that children and adults expend significantly more energy during gameplay than rest [16, 17]. It is important to note that video games (previously described) are different to esports, as video games are designed to be played leisurely, whereas esports are designed to be played competitively.

Only three studies have investigated the energy expenditure of esports within amateur e’athletes, yielding conflicting results [18–20]. A case report by Haupt et al. [18] and findings from Zimmer et al. [19] showed that esports gameplay did not significantly increase V̇O_2_, V̇CO_2_, RER, or energy expenditure when compared to rest, and even suggested that gameplay did not result in a stress response when analysing blood lactate, cortisol, and glucose responses [19]. Whereas, Kocak [20] showed that amateur level *League of Legends* e’athletes expend 40% more energy or 1.9 METs during gameplay when compared to rest, which could be classified as light physical activity. While this research provides valuable insight into the physiological demands of esports and video gaming, there are recurrent methodological limitations; for instance, failure to control for variables that affect energy expenditure (i.e., caffeine or alcohol intake, diet, environmental conditions, sleep, transport, physical activity, wellness, fatigue, etc.), poorly defined resting conditions, and sample amateur e’athletes. Compher et al. [21] have identified the importance of control variables when measuring metabolic rate, which should be followed within future esports research for accurate interpretation and to enable comparisons across findings. The gameplay conditions presented in Haupt et al. [18] and Zimmer et al. [19] were limited by non-competitive environments, and research needs to investigate the metabolic demands of expert e’athletes within competitive environments. Currently, there is a lack of physiological research in esports, and the limitations described highlight that the current research lacks methodological consideration. The physiological demands of competitive esports are poorly understood, and further research is necessary to support the health and wellbeing of e’athletes.

Heart rate variability (HRV) recording is non-invasive, low-cost, and simple to administer, with most of the physiological research in esports using this method to assess autonomic regulation during the activity. Within applied settings, HRV can also provide insight into an individual’s ability to cope with internal and external stressors, where low HRV is associated with impaired ANS regulation [22]. Research within esports has shown a significant decrease in R-R interval reflecting an increase in heart rate during gameplay when compared to rest [23, 24]. Additionally, e’athletes within winning teams have demonstrated a significantly higher percentage of successive normal sinus RR intervals more than 50ms (pNN50) and root mean square of successive differences (RMSSD) measurements post-game, when compared to losing teams, which has been hypothesised to be due to poor self-regulation or greater recovery [25, 26]. RMSSD and pNN50 are time-domain measurements which are used to estimate the vagally mediated changes in HRV [27]. Other research has added to these findings, stating that HRV is susceptible to inter-participant variability in results [23, 24], which is hypothesised to be due to different games, in-game roles, or physiological differences in body composition and physical activity levels. Inter-participant differences in HRV could also be explained by the individual’s appraisal of various internal and external stressors during gameplay [22], which can be different from training to competition [28]. Additionally, Welsh et al. [29] have identified that HRV research within esports currently lacks theoretical underpinning and a lack of methodological consideration, identifying that further investigation is needed within esports.

The study aimed to quantify individual RMR and compare it to gameplay metabolic rate (GMR), whilst simultaneously measuring HRV to investigate autonomic regulation during competitive gameplay within expert e’athletes. We hypothesised that GMR would be significantly higher than RMR across the participant group while decreasing markers of HRV and vagal tone during gameplay.

More specifically, the study aimed to answer the following research questions:

1. How does competitive esports gameplay affect metabolic rate within expert e’athletes?
2. How does competitive esports gameplay affect HRV among expert e’athletes?
3. What is the relationship between HRV and metabolic rate during rest and gameplay among expert e’athletes?

## Methodology

Participants were recruited from a local intervarsity esports academy, which competes within the national intervarsity league for various game titles, with some individuals also competing within professional leagues. Eligibility criteria allowed for participants to play any game title based on PC, with participants required to be ranked within the top 10% of their game, which equates to > Diamond (*Overwatch*), > Diamond 2 (*Valorant*), > Champion 1 (*Rocket League*), > Platinum 4 (*League of Legends*), and > Legendary Eagle (*Counter-Strike: Global Offensive*). Participants also had to be apparently healthy, with no acute or chronic conditions known to affect metabolic rate or cardiac response, and no consumption of medications that affect metabolic rate. Physical activity levels were accounted for within the control measure questionnaire, with participants totalling their weekly activity minutes. A priori sample size calculation was performed (G*power software version 3.1.9.6) through a one-tailed t-test, using Cohen’s *d* = 0.75 determined from previous research [30], α of 0.05, and a power of 0.8, resulting in the study requiring 13 participants.

EGAIC was measured through a silicone mouthpiece (Hans Rudolph Adult Silicone Mouthpiece) hosting a T-valve where a 3-L latex mixing bag modified with a 2 cm diameter circular inferior exhaust opening to allow airflow from the bag at higher tidal volumes was fitted to the expired side of the mouthpiece. The custom expired mixing bag allowed for variable volume performance of the mixing bag, and details of the device and its validation have been previously published [31]. The mixing bag contained a gas sample line that allowed gas sampling from the upper central region of the bag (Tygon Tubing, ID = 2.8 mm, OD = 3.9 mm; Fisher Scientific Company, Pittsburgh, Pa., USA), which was connected to a set of electronic CO_2_ and O_2_ analysers (AEI Technologies) and gas flow pump. Expired gas signals of the prior breath were acquired for 100ms at the start of each inspired breath and aligned to the timing of the start of inspiration based on a pre-determined measured time-delay. On the inspired side of the mouthpiece, ventilation was measured by an infrared flow-turbine (UVM, VacuMed, Ventura, CA, USA). Data acquisition was performed through custom made LabView software (LabVIEWTM, Austin, TX), and commercial electronic acquisition devices (National Instruments, Austin, TX). This custom software has been validated within previous studies and validated against commercial systems [31].

Prior to data collection for each participant, the gas analysers and flow turbine were calibrated. This was performed using the custom-developed LabVIEW software (LabVIEW, National Instruments, Austin, TX, USA) through a computerised custom-developed data acquisition system (National Instruments). Before all tests, the gas analysers were calibrated with medical grade and certified calibration gas (3.2% CO_2_, 16.2% O_2_, balance N_2_), room air (20.95% O_2_, 0.04% CO_2_, balance N_2_), and 100% nitrogen, with regressions calculated within the software. Turbine calibration was performed using a 3-L calibration syringe (Hans Rudolph, Kansas City, Mo., USA). The EGAIC system and methodology is discussed in more detail by Kim and Robergs [31].

The ECG equipment (Custo-Cardio 300) was fitted to each participant using a standardised 5-lead ECG configuration. The ECG leads were attached to the participants using gel electrodes placed over the spine of both scapulae, the iliac crest of both ilia, and between the 4th and 5th intercostal space along the mid-axillary line of the left side of the torso. The Custo-diagnostic software converted the cardiac electrical signal from the heart to R-R intervals during each condition. This system was connected via Bluetooth to a dedicated laptop running software (Custo-Diagnostic Software, Custo-Med Gmbh), with the device attached to the participant. ECG data was visually inspected for technical artefact, and abnormal R-R intervals were removed from the dataset. Technical artifact may result from excessive movement of participant, or signal disruption at the electrode-skin connection, as the R peak duration consistently differs from real heartbeats. Visual inspection of ECG data is recommended over using automatic correction filters in *Kubios*, as they can lead to the removal of real heartbeats [32]. Data were also inspected for physiological artefact, such pre-ventricular contraction, and fibrillations, however these were not identified within the included participants. As the testing environment and experimental condition required minimal movement of the participant, data was relatively clear of technical artifact. Excel files derived from the ECG software (.csv) were converted into text files (.txt) and processed within HRV analysis software, *Kubios* [33]. This software was used to calculate the following HRV parameters: RMSSD and pNN50 within the time domain, and high frequency (HF) and low/high frequency ratio (LF/HF), as they best represent vagal tone [32] Table 1.

**Table 1:** HRV variables that were collected and derived from Laborde et al. [26]

Domain	Variable	Description	Physiological origin
Time-domain	RMSSD	Root Mean Square of Successive Differences	Vagal Tone
	pNN50	Percentage of successive normal sinus RR intervals more than 50ms	Vagal Tone
Frequency-domain	HF	High Frequencies	Vagal Tone
	LF/HF	Low Frequencies/ High Frequencies Ratio	Mix of sympathetic and vagal activity

### Procedures

E’athletes were instructed to attend the laboratory soon after waking, avoiding physical activity (transport via bus or car). Prior to attendance, participants were informed that they needed to be fast for at least 5 h before testing, at least 24 h abstention from alcohol, 2 h from nicotine, 8-hours abstention from caffeinated products, as well as refraining from moderate-high intensity exercise performed 24 h before data collection [21]. Participants were also instructed to sleep for at least 7 h before data collection, as it has been shown that achieving 6 h or less of sleep could impair cognitive and physical performance [34]. Height and weight were measured, and body mass index (BMI) was calculated. All participants were asked to complete a control measures questionnaire to ensure adherence to pre-testing instructions (see Additional File 1: Table 1). This testing session was rescheduled if participants did not adhere to control measures. This questionnaire also contained the Stafford Sleepiness Scale [35] to assess the level of sleepiness, and a wellness questionnaire which asked the participant to rate their levels of fatigue, sleep quality, general muscle soreness, stress levels, and mood on a Likert scale from one to five [36]. The ESSA Adult Pre-Exercise Screen Tool [37] was used to identify any major health concerns, and collect overall weekly physical activity duration. The physical activity duration was used to determine if participants reached the World Health Organisation (WHO) physical activity guidelines of 150 min of moderate-to-vigorous aerobic exercise a week [38]. A question was also added regarding medication usage; if an individual indicated that they consumed a medication that affected metabolic rate or answered ‘yes’ to any of the first six questions, they were excluded from the study.

The participant was fitted with the 5-lead ECG and Hans-Rudolph silicone mouthpiece to assess RMR and resting HRV. They were instructed to sit quietly and upright in their regular gaming chair. The testing environment was temperature-controlled, quiet, and dimly lit. The participant sat quietly, instructed not to talk, for a total of 20 min [21].

Immediately following the resting condition, the participant loaded into a solo-queued ranked game. The participant was seated within the same chair as the resting condition and fitted with the Hans Rudolph Mouthpiece (Hans Rudolph, Kansas City, Mo., USA). The start of all game titles was defined as the moment they were in the game. For *League of Legends*, this point was defined as after the champion select and when the character entered summoners rift. For *Valorant* and *Counter Strike: Global Offensive* this was defined as when the character could buy. For *Rocket League*, this was defined as when the count-down into the game had finished, and the 5 min round had started. Due to the mouthpiece configuration, e’athletes were instructed to use in-game ‘pings’ to communicate and were instructed to avoid attempting to talk.

The raw breath-by-breath data was exported as an csv. File into commercial graphics fitting software, Prism GraphPad (Prism, GraphPad Software, La Jolla, CA, USA). The raw data included the absolute values for V̇O_2_, V̇CO_2_, RER, VE and two measures of energy expenditure. The software’s energy expenditure calculation converted the RER measure into caloric equivalent and this was multiplied by the corresponding V̇O_2_ value. The Peronnet and Massicotte [39] equation for energy expenditure was also calculated for each breath as it is shown to have the greatest metabolic power [40]. The first and last 5 min periods of the RMR data were deleted, and the middle 10 min was used [21].

For GMR, the HRV and EGAIC data was cut from game start to end as defined earlier for each game. Errant breaths, swallows, or coughs were first removed from the EGAIC data to not skew the response. These were identified as breaths that were different to the mean of the surrounding four data points by more than three times the standard deviation of those four points [41–43]. The HRV and EGAIC data was averaged and analysed for the entire game duration for each title (*Rocket League* ≈ > 5 min, *League of Legends* ≈ 25–45 min, *Counter-Strike: Global Offensive* ≈ 35 min, and *Valorant* ≈ 25 min).

Data analysis was performed within Jamovi [44, 45]. All demographic information was expressed as means, standard deviations and frequencies. The physiological data was assessed for normality, where it was shown to be non-parametric. The data were then presented as medians and 25th and 75th percent interquartile ranges. To answer the first two research questions, the Wilcoxon signed-rank test was used as the data meets the three assumptions for this test. The third research question used a Spearman correlation matrix to investigate the relationship between all outcome variables for rest and gameplay. Significance was identified when *p* ≤ .05.

## Results

A total of 13 male participants completed the project and their demographic characteristics and individual characteristics and metabolic changes to each condition are presented in Tables 2 and 3, respectively.

**Table 2:** Demographic characteristics of participants

	Participants, *n* = 13 (Mean ± SD)
Age (years)	20.7 ± 2.69
Height (cm)	183 ± 7.89
Weight (kg)	82.1 ± 18.6
BMI (kg·m^− 2^)	24.6 ± 5.89
	***N (%)***
Reaching WHO PA Guidelines	4 (30.8)
**Game Title**	
Rocket League	5 (38.5)
League of Legends	4 (30.8)
Overwatch	2 (15.4)
Valorant	1 (7.7)
Counter Strike: Global Offensive	1 (7.7)

*Note* SD = standard deviation; Reaching WHO PA Guidelines = Number of participants exceeding 150 min of moderate-to-vigorous physical activity a week

**Table 3:** Individual characteristics of metabolic changes between resting and gameplay metabolic rates

ID#	BMI	Game	Game genre	Resting metabolic rate						Gameplay metabolic rate					
				VE	V̇O_2_	V̇CO_2_	RER	EE	PM EE	VE	V̇O_2_	V̇CO_2_	RER	EE	PM EE
1	19.9	LOL	MOBA	5.25	4.07	3.36	0.83	1.12	4.84	7.63	6.19	4.10	0.66	1.66	7.09
2	24.7	OW	FPS	12.63	3.69	3.66	0.87	1.53	6.86	8.58	4.49	2.85	0.66	1.77	7.41
3	30.6	OW	FPS	10.96	3.85	3.19	0.85	1.94	8.34	12.38	4.60	4.12	0.89	2.34	9.90
4	20.7	LOL	MOBA	6.29	3.25	2.52	0.80	1.17	5.05	7.32	3.13	2.98	0.98	1.14	4.94
5	18.9	RL	Sports	11.95	5.15	5.27	1.00	1.54	6.68	7.99	4.05	4.36	1.10	1.20	5.20
6	19.5	RL	Sports	10.26	4.31	3.48	0.82	1.47	6.33	10.28	3.90	3.56	0.92	1.35	5.83
7	33.1	CSGO	FPS	7.71	2.72	2.44	0.90	1.49	6.43	9.63	3.22	2.97	0.91	1.78	7.66
8	29.1	RL	Sports	5.11	2.69	1.92	0.73	1.16	5.00	11.38	4.98	4.14	0.85	2.17	9.36
9	22.2	RL	Sports	10.26	2.33	2.32	0.92	1.02	4.51	8.71	3.28	3.06	0.92	1.43	6.18
10	19.0	LOL	MOBA	6.70	3.55	3.05	0.87	1.16	5.02	8.18	3.48	3.12	0.87	1.14	4.99
11	32.5	RL	Sports	5.95	2.77	2.14	0.77	1.39	5.99	8.38	2.82	2.57	0.90	1.45	6.26
12	18.2	LOL	MOBA	4.75	2.59	2.33	0.88	0.78	3.36	5.73	3.34	3.18	0.95	1.02	4.37
13	31.9	VL	FPS	6.65	2.88	2.06	0.75	1.28	5.56	8.70	3.13	2.96	0.94	1.50	6.38

*Note* BMI = Body Mass Index; LOL = League of Legends; OW = Overwatch; RL = Rocket-League; CSGO = Counter Strike: Global Offensive; VL = Valorant; MOBA = Multiplayer Online Battle Arena; FPS = First Person Shooter; Sports = Sports Simulation; VE = Ventilation; V̇O_2_ ml/kg = Oxygen Consumption; V̇CO_2_ ml/kg = Carbon Dioxide Production; RER = Respiratory Exchange Ratio; EE = Energy Expenditure; PM EE = Peronnet and Massicotte energy expenditure

**Fig. 1:**
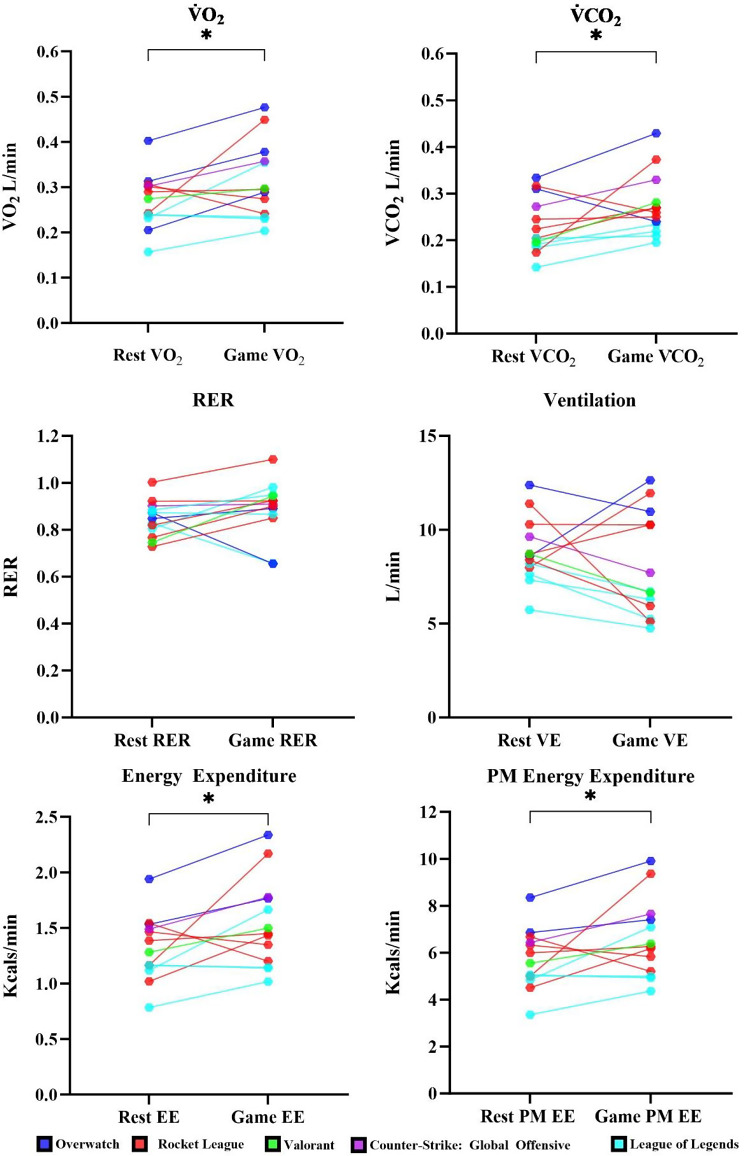
Participant median values for EGAIC variables between rest and gameplay conditions

**Fig. 2:**
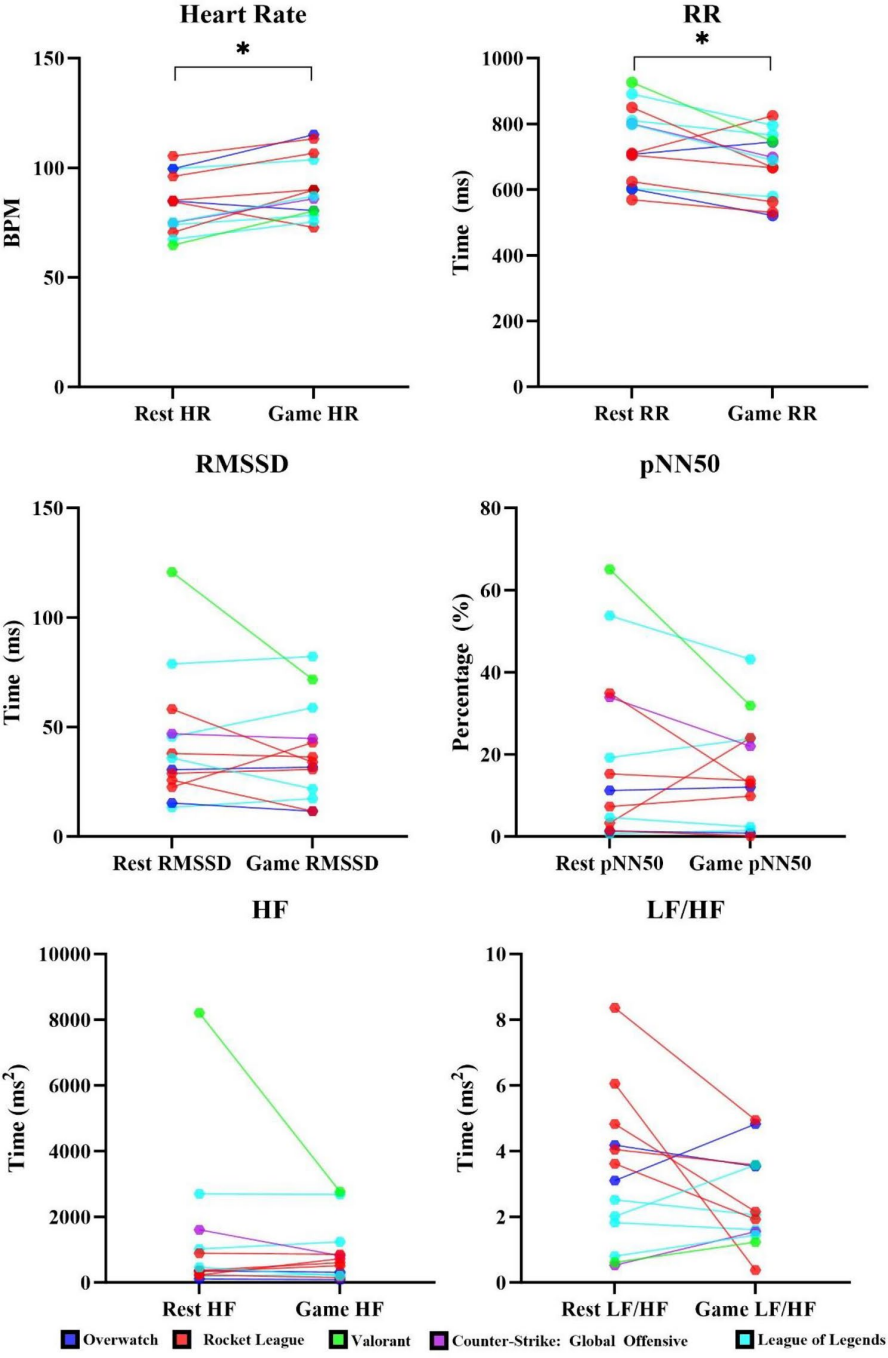
Individual median values for HRV variables between rest and gameplay conditions

Table 4 below presents the descriptive statistics for EGAIC metabolic rates and HRV variables and the Wilcoxon signed ranked paired t-test results. For EGAIC, all values showed significant differences with moderate to large effect sizes, apart from VE and RER. For HRV, HR and RR were significantly different with large effect sizes.

**Table 4:** Descriptive statistics and analysis of group EGAIC and HRV between resting metabolic rate and gameplay metabolic rate

*N* = 13	Resting metabolic rate, median (IQR 25–75)	Gameplay metabolic rate, median (IQR 25–75)	*p*-value	Effect size
**EGAIC variables**				
VE (L/min)	6.70 (5.95–10.3)	8.58 (7.99–9.63)	0.13	− 0.36
V̇O_2_ (L/min)	0.27 (0.24–0.30)	0.29 (0.24–0.35)	0.02*	− 0.60
V̇CO_2_ (L/min)	0.20 (0.19–0.27)	0.27 (0.24–0.33)	0.01*	− 0.67
RER	0.84 (0.80–0.88)	0.91 (0.86–0.94)	0.08	− 0.45
EE (kcal/min)	1.28 (1.16–1.49)	1.45 (1.20–1.77)	0.02*	− 0.64
PM EE (kcal/min)	5.55 (5.00–6.43)	6.26 (5.20–7.41)	0.02*	− 0.64
**HRV variables**				
HR (bpm)	84.5 (74.1–96.1)	87.1 (80.3–104)	0.01*	− 0.74
RR (ms)	710 (624–810)	689 (579–747)	0.02*	0.71
RMSSD (ms)	35.9 (25.7–46.9)	34.0 (21.8–44.8)	0.49	0.23
pNN50 (%)	11.2 (3.32–33.9)	12.9 (2.36–23.8)	0.34	0.32
HF (ms^2^)	368 (230–1022)	603 (254–856)	0.73	0.12
LF/HF (ms^2^)	3.10 (1.82–4.18)	2.06 (1.55–3.57)	0.45	0.25

Note*= significance (Wilcoxon test- *p* < .05). VE = ventilation; V̇O_2_= oxygen consumption; V̇CO_2_= carbon dioxide elimination; RER = respiratory exchange ratio; EE = energy expenditure derived from RER and V̇O_2_; PM EE = Peronnet and Massicotte energy expenditure; HR = Heart Rate; RMSSD = root mean square of successive differences; pNN50 = Percentage of successive normal sinus RR intervals more than 50ms; HF = High frequency; LF/HF = Low to High frequency ratio

Tables 5 and 6 display the results of a Spearman correlation matrix for both resting and gameplay measures for HRV and EGAIC variables. Both tables demonstrate no correlation between any HRV or EGAIC variables measured for either resting or gameplay conditions.

**Table 5:** Spearman correlation matrix for resting measures

	Rest VE	Rest V̇O_2_	Rest V̇CO_2_	Rest RER	Rest EE	Rest PM EE	Rest HF/LF	Rest HF	Rest HRV pNN50	Rest HRV RMSSD	Rest mean RR	Rest mean HR
Rest VE	—											
Rest V̇O_2_	0.71^**^	—										
Rest V̇CO_2_	0.88^***^	0.86^***^	—									
Rest RER	0.48	0.01	0.40	—								
Rest EE	0.71^**^	0.98^***^	0.86^***^	0.04	—							
Rest PM EE	0.75^**^	0.98^***^	0.87^***^	0.06	0.99^***^	—						
Rest HF/LF	0.06	0.20	0.19	-0.27	0.17	0.11	—					
Rest HF	-0.31	-0.34	-0.47	-0.15	-0.35	-0.32	-0.64^*^	—				
Rest HRV pNN50	-0.18	-0.19	-0.35	-0.26	-0.23	-0.20	-0.43	0.95^***^	—			
Rest HRV RMSSD	-0.25	-0.28	-0.42	-0.17	-0.31	-0.29	-0.51	0.97^***^	0.98^***^	—		
Rest Mean RR	-0.40	-0.33	-0.57^*^	-0.40	-0.37	-0.34	-0.51	0.92^***^	0.88^***^	0.89^***^	—	
Rest Mean HR	0.40	0.33	0.57^*^	0.40	0.37	0.34	0.51	-0.92^***^	-0.89^***^	-0.89^***^	-1^***^	—

*Note* * *p* < .05, ** *p* < .01, *** *p* < .001

**Table 6:** Spearman correlation matrix for gameplay measures

	Game VE	Game V̇O_2_	Game V̇CO_2_	Game RER	Game EE	Game PM EE	Game HRV HF/LF	Game HF	Game HRV pNN50	Game HRV RMSSD	Game mean RR	Game mean HR
Game VE	—											
Game V̇O_2_	0.72^***^	—										
Game V̇CO_2_	0.84^***^	0.76^**^	—									
Game RER	-0.32	-0.68^*^	-0.14	—								
Game EE	0.74^**^	1^***^	0.79^**^	-0.64^*^	—							
Game PM EE	0.74^**^	1^***^	0.79^**^	-0.64^*^	1^***^	—						
Game HRV HF/LF	-0.17	-0.07	-0.22	-0.08	-0.11	-0.11	—					
Game HF	0.09	-0.14	-0.01	0.04	-0.12	-0.12	-0.66^*^	—				
Game HRV pNN50	0.14	-0.24	-0.12	0.06	-0.22	-0.22	-0.48	0.92^***^	—			
Game HRV RMSSD	0.09	-0.23	-0.10	0.07	-0.20	-0.20	-0.52	0.95^***^	0.98^***^	—		
Game Mean RR	-0.08	-0.37	-0.43	0.09	-0.38	-0.38	-0.34	0.74^**^	0.87^***^	0.84^***^	—	
Game Mean HR	0.08	0.37	0.43	-0.09	0.38	0.38	0.34	-0.74^**^	-0.87^***^	-0.84^***^	-1^***^	—

*Note* * *p* < .05, ** *p* < .01, *** *p* < .001

## Discussion

The aim of the study was to investigate the metabolic demands and HRV during esports gameplay. The hypothesis was supported with significantly higher metabolic rates displayed within the GMR conditions across the group when compared to the RMR condition. For HRV variables, heart rate (and by definition, RR-intervals) were the only significantly different variables across conditions, which does not support the original hypothesis.

Our results demonstrated that e’athletes expended significantly more energy during solo-queued ranked gameplay compared to rest. Differences in energy expended between solo-queue and rest were identified through increases in V̇O_2_, V̇CO_2_, and both measures of EE, supported by large effect sizes. Our results showed a 17% increase in EE during gameplay, which is less than half the game-play induced increase when compared to previous research that showed multiplayer online battle arena (MOBA) players expend up to 40% more energy during gameplay [20]. Increases in energy expenditure can be partly explained through an increase in activity, where Kocak [20] demonstrated a strong significantly positive correlation between actions per minute and MET’s. The increases in EE in the current investigation are different from previous findings, which showed that esports gameplay does not affect energy expenditure, O_2_ consumption, or CO_2_ production among amateur Counter-Strike and FIFA players [18, 19]. Differences across studies could be due to our participants being expert e’athletes when compared to the amateur participants within previous studies and different methodologies used as our study used a controlled resting condition for comparison to gameplay. These findings suggest that higher level e’athletes, different game title, or more competitive environments may elicit higher metabolic rates, however this requires further investigation.

The median RER value for the RMR condition was 0.84 (0.80–0.88), and increased to 0.91 (0.86–0.94) during the GMR condition, suggesting a potential increase in glucose oxidation during sedentary activity. Notably, these findings did not reach statistical significance in our small sample, and the observed 0.84–0.93 shift may be subject to chance variation. However, an increase in RER of this magnitude is not generally observed within sedentary activities that involve minimal skeletal muscle recruitment. Hyperventilation, a common factor observed in laboratory testing settings due to participant anxiety, was not observed within either condition, and the increase in RER may be due to greater carbohydrate oxidation during gameplay. The observed RER results are different from previous studies by Zimmer et al. [19] and Troubat et al. [46] who observed a significant time effect on RER, with significantly lower values in the post-phase of gaming. When converting the RER values observed within our study into a percentage of energy derived from carbohydrate oxidation, differences across conditions were 48.3% carbohydrate oxidation in the RMR to 71.9% carbohydrate oxidation in the GMR condition [39]. This is supported by previous findings where a cognitively demanding incongruent Stroop task elicited a significantly higher RER when compared to a congruent Stroop and rest conditions [47].

Increased utilisation of carbohydrates as the primary fuel source is generally seen when exercise intensity increases and there is high skeletal muscle motor unit recruitment (e.g., walking into high-intensity running). However, within the GMR condition, participants were seated while playing their respective esports title, which may indicate the increase in carbohydrate oxidisation within the GMR condition could be due to the increase in neural activity. This is a plausible explanation as the brain relies on glucose as the main energy supply and accounts for 20% of glucose metabolism in the body [48, 49]. Evidence also supports this interpretation, as it highlights results from prior research of significant decreases in blood glucose levels under increased neural activity, where greater mental load and task complexity increases the demand for glucose [50, 51]. Neuroimaging through positron emission tomography, has shown that visual stimulation, mental activity, and exhaustive exercise significantly decrease the oxygen-glucose index when compared to rest, highlighting the higher utilisation of glucose during these tasks [52–55]. Future research into the physiological demands of esports may incorporate neuroimaging or blood testing to further investigate the effect of esports gameplay on cerebral metabolic rate.

Exercise training within e’athletes may influence substrate metabolism during competitive gameplay. The training status of individuals affects substrate metabolism during exercise and contributes to the rate of carbohydrate metabolism during activity [56, 57]. Studies have shown a greater utilisation of lipids during moderate intensity exercise after endurance training [58], which may apply to tasks of increased cognitive load. Exercise training also induces positive mental health, improved cognitive function, and greater adaptability to various stressors [59]. Therefore, it could be hypothesised that specific exercise training could indirectly enhance esports performance and overall health of e’athletes, although further investigation is needed to support this.

There was a significant increase in heart rate and decrease in RR-intervals from RMR to GMR with large effect sizes. However, no measure of HRV displayed significant differences between conditions, which could indicate that solo-queued ranked gameplay across game titles does not elicit a stress response. Previous findings have highlighted that live esports competition generated vagal mediated responses, reflected through a significant decrease in HF power [60], which could be explained by e’athletes reporting stressors within competition are more intense than team or solo training [28]. This could explain why the results observed in our study are different to those obtained during live competitive games, as e’athletes experience greater stressors during competitive games when compared to playing a solo-queued ranked game. E’athletes with more experience have been shown to have higher HRV values when compared to casual e’athletes in the end-phase of gaming, which indicates that expert e’athletes are able to cope with stress better in the late game [61]. The participants in the current study were expert e’athletes, which may explain why no differences were observed for any HRV variables between rest and gameplay, as they may not perceive solo-queued ranked gameplay as a stressor. Additionally, e’athletes within winning teams have demonstrated significantly higher pNN50 and RMSSD measurements post-game, when compared to losing teams, which has been hypothesised to be due to poor self-regulation or greater recovery [25, 26].

When compared to normative ranges within healthy subjects, the resting R-R interval, RMSSD, pNN50 and HF measurements within this study are lower [62, 63]. Lower HRV values can be indicative of an increased risk of cardiovascular disease- related morbidity and mortality [64, 65]. Lower HRV observed within this study may also be explained by the physical activity behaviour reported by the group, as the majority (69.2%) did not meet the WHO physical activity guidelines [38]. Exercise training has been shown to elevate HRV values after significant improvements in aerobic fitness, and mitigate the age-related decline in HRV [66]. Additionally, there is a correlation between lower HF power and stress, panic, anxiety, or worry [67]. Control measure questionnaires prior to data collection did not identify extreme levels of stress, sleepiness, or mood prior to testing, which may indicate that the lower levels of HRV could be due to poor physical activity behaviours. This finding adds some support to implementing physical exercise training and promoting physical activity within the esports industry.

This study presents with multiple strengths and limitations. The first strength of this study is that the sample size met the prior sample size calculation which provided sufficient power during data analysis. Meticulous methodology and sensitive equipment were then used to acquire data and thereby minimise instrumentation and researcher errors. This, in turn, improved the power of the research study and related statistical analyses (minimised type-2 errors). This study also adds to the literature as it is one of the first studies to use EGAIC within expert e’athletes, which provided further insight into the physiological demands of esports changes to whole-body metabolism.

Limitations within the study include using solo-queued ranked games instead of more important matches, such as team scrimmages or live competition, which has been identified in the literature as more stressful than solo or team training [28]. The EGAIC equipment is a major distraction within gameplay and far from the normal environment. This means that e’athletes and coaches were only comfortable playing solo-queued ranked games under testing conditions. Less invasive methodologies will need to be used to gain access to players under real competition conditions, or to develop research methods that effectively provide a real-game scenario, perhaps through the provision of meaningful prizes or other forms of reward. Another limitation of the study is the under representation of females within the sample. This is an area for future research as a lack of female participation is a consistent problem within the industry [68]. The methodology used within this project lacks theoretical underpinning, which Welsh et al. [29] has identified as a limitation within esports. Recruiting local e’athletes ranked within the top 10% of their game to participate within a laboratory-based project is challenging. Due to participant recruitment difficulties, participants were grouped together as ‘e’athletes’ across four different esport titles, which may affect the results. Future research needs to investigate the differences across game titles, and the potential influence of certain in-game roles.

## Conclusion

In conclusion, the findings demonstrated that solo-queued ranked gameplay significantly increased energy expenditure, O_2_ Consumption, and CO_2_ production when compared to rest. Increases in energy expenditure are believed to be due to the increase in cognitive load during gameplay as the activity is performed in a seated position. The findings also demonstrated that HR was significantly increased during gameplay, although all markers of HRV showed no significant difference, which may indicate that solo-queued gameplay does not elicit a significant stress response. Resting values of HRV were lower than normative values within healthy subjects, which is a major concern as this indicates an increased risk of cardiovascular-related morbidity and mortality. The results of this study provide further insight into the physiological demands of competitive esports gameplay; however, further investigation is needed within competitive environments.

### Electronic Supplementary Material

Below is the link to the electronic supplementary material.

Supplementary Material 1

## Data Availability

The datasets used and/or analysed during the current study are available from the corresponding author on reasonable request.

## Abbreviations

VO_2_: Oxygen Consumption
CO_2_: Carbon Dioxide Production
EE: Energy Expenditure
RER: Respiratory Exchange Ratio
EGAIC: Expired Gas Analysis Indirect Calorimetry
HRV: Heart Rate Variability
ANS: Autonomic Nervous System
RMSSD: Root Mean Square of Successive Differences
pNN50: Percentage of successive RR intervals that differ by more than 50ms
HF: High Frequency
LF/HF: Low and High Frequency Ratio
